# Nootkatone mitigates LPS-induced acute lung injury by modulating the NF-κB and Nrf2 pathways in mice

**DOI:** 10.3389/fvets.2025.1593919

**Published:** 2025-11-06

**Authors:** D. D. V. Hanuman, Anil Kumar Banothu, Kala Kumar Bharani, Ravi Kumar Yadala, Amit Khurana, Ambica Gadige, Nagarjuna Gandham

**Affiliations:** 1 Department of Veterinary Pharmacology and Toxicology, College of Veterinary Science, P.V. Narsimha Rao Telangana Veterinary University, Hyderabad, India; 2 Department of Veterinary Pathology, College of Veterinary Science, P.V. Narsimha Rao Telangana Veterinary University, Hyderabad, India; 3 Department of Veterinary Medicine, College of Veterinary Science, P.V. Narsimha Rao Telangana Veterinary University, Hyderabad, India

**Keywords:** nootkatone, lipopolysaccharide, acute lung injury, inflammation, oxidative stress

## Abstract

Acute lung injury is a serious condition that can be life-threatening. Nootkatone, a promising phytochemical, possesses notable antioxidant and anti-inflammatory properties. The current study aimed to investigate the anti-inflammatory effects of nootkatone both *in vitro* using Raw 264.7 macrophages and *in vivo* through intraperitoneal administration at doses of 50 and 100 mg/kg for 7 days. Animals were divided into five groups: normal control (NC), disease control (DC; LPS 10 μg/kg, oropharyngeal), nootkatone low dose (NLD; 50 mg/kg, i.p. for 7 days + LPS), nootkatone high dose (NHD; 100 mg/kg, i.p. for 7 days + LPS), and nootkatone per se (NPS; 100 mg/kg nootkatone alone). The study assessed the efficacy of nootkatone against lipopolysaccharide (LPS)-induced lung injury in mice, with LPS administered via the oropharyngeal route at 10 μg/kg. Concentrations of nootkatone up to 50 μM were found to be safe, showing only a modest reduction in cell viability. Treatment with various doses of nootkatone effectively mitigated LPS-induced inflammation in Raw 264.7 macrophage cells, as indicated by modulation of inflammatory cytokines such as IL-10 and TNF-*α*. We observed significant (*p* < 0.05) alteration in absolute and relative lung weights, as well as in hematological profiles. The levels of cytokines (IL-6, TNF-*α*, IL-1β, IL-22; IL-17; IFN-*γ*; TGF-β1, and IL-10) were significantly modulated in the NLD (50 mg/kg) and NHD (100 mg/kg) groups. Furthermore, the levels of malondialdehyde and nitrite were significantly lower in these groups compared to the disease control. Histological analysis revealed the reversal of lung tissue damage in the treated group compared to the disease control group. Notably, immunohistochemical evaluation of Nrf-2, NF-κB, TNF-*α,* and COX-2 expression further confirmed the potent anti-inflammatory effects of nootkatone. This study provides comprehensive evidence that nootkatone may represent a promising candidate for further research in the management of pulmonary inflammation.

## 1 Introduction

Acute lung injury (ALI) and its more severe form, acute respiratory distress syndrome (ARDS), are among the most devastating medical conditions and have been extensively studied over the past 50 years. The pathophysiological effects of ALI/ARDS, such as impaired gas exchange, alveolar collapse, and hypoxia, are further aggravated by bilateral thoracic edema. These conditions predominantly affect young, otherwise healthy people and cause thousands of deaths among adults and children worldwide each year (1). Importantly, ALI is also recognized in veterinary medicine. Companion animals such as dogs and cats may develop ALI secondary to pneumonia or sepsis, while horses and neonatal foals often present with sepsis-related respiratory distress. Similar conditions have been reported in livestock exposed to infectious or toxic agents. Because current treatment options remain primarily supportive, identifying novel therapeutic agents such as nootkatone could have significant translational value for both human and veterinary medicine. In patients with predisposing risk factors, the incidence of ALI and ARDS is reported to be 32.7 and 30%, respectively (2). In a comparative study, Murray’s criteria revealed an incidence rate of 25%, whereas the North American–European Consensus Conference (NAECC) criteria reported a 41.8% incidence and an 11% hospital fatality rate for ARDS and ALI, respectively (3).

Extensive research has been conducted to identify the causative agents, epidemiology, and pathogenesis of ALI, as well as to develop suitable treatment strategies leveraging advancements in intensive care unit (ICU) life-support systems. Lipopolysaccharide (LPS), a major component of the outer membrane of Gram-negative bacteria, is a significant airborne contaminant that contributes to multiple organ failures, sepsis, and pneumonia. It is recognized as one of the most common etiological factors of ALI. LPS is a powerful inducer of inflammatory responses through the direct modulation of the immune system (4).

Toll-like receptor-4 (TLR-4), located on epithelial cells and alveolar macrophages, recognizes LPS during pulmonary infection and thus plays an important role in pulmonary innate immunity (5). When LPS binds, multiple intracellular signaling cascades are activated, leading to the translocation of transcription factors into the nucleus and the release of several important proinflammatory mediators. Moreover, adhesion molecules are upregulated within the pulmonary vasculature (6). Activated neutrophils subsequently release proteases, oxygen-free radicals, prostaglandins (PGs), and prostacyclins (PIs), which damage alveolar epithelial and capillary endothelial cells, as well as alveolar wall ion channels, resulting in irreversible flooding of the alveolar lumen.

Despite the availability of several treatment methods, instances of ALI/ARDS are currently treated primarily with supportive care due to a lack of specialized chemotherapeutic options and the risk of bacterial resistance. Nootkatone (NTK), a ketone sesquiterpenoid and the major component of grapefruit, belongs to the largest subclass of terpenes. It is also found in *citrus*, *Alpinia*, *Chrysopogon*, and other genera of essential oils, as well as in aromatic plants such as Alaskan cedar and Java cedar. Previous studies have demonstrated the anti-cancer, anti-fibrotic, antioxidant, anti-apoptotic, hepatoprotective, nephroprotective, neuroprotective, and dyslipidemia-modulating properties of NTK. *In vitro* studies have also examined its anti-inflammatory effects in LPS-induced neuroinflammation, primarily through the regulation of the AMP-activated protein kinase (AMPK) signaling pathway (7). However, the effects of NTK on LPS-induced ALI/ARDS in animal models have not been studied.

Given its well-known anti-inflammatory and antioxidant properties, we hypothesized that NTK could mitigate LPS-induced lung damage. To test this, we employed a non-invasive and highly reproducible mouse model of lung damage produced by LPS instillation via the oropharyngeal route. The protective benefits of NTK were assessed through the analysis of bronchoalveolar lavage (BAL) fluid parameters, including cellular profiles such as macrophages, neutrophils, and lymphocytes. Detailed investigations were further conducted using biochemical assays to assess oxidative stress, cytokine measurements to evaluate inflammatory responses, histological examination, and immunohistochemical analysis of selected inflammatory markers.

## 2 Materials and methods

### 2.1 Chemicals and reagents

Nootkatone was purchased from Tokyo Chemical Industry (TCI), Japan (Catalog no. N0120; purity ≥97%). LPS from *Escherichia coli* (B1:055), Griess reagent, sodium dodecyl sulfate (SDS), and glacial acetic acid (GAA) were purchased from Sigma-Aldrich, MA, USA. Enzyme-linked immunosorbent assay (ELISA) kits for interleukin-6 (IL-6), tumor necrosis factor-*α* (TNF-α), IL-1β, IL-22, IL-17, interferon-*γ* (IFN-γ), transforming growth factor-β1 (TGF-β1), and IL-10 were procured from Invitrogen (Thermo Fisher Scientific, USA). The immunohistochemistry kit was purchased from Pathnsitu Biotechnologies, Hyderabad, India. Ellman’s reagent (5,5′-dithiobis-(2-nitrobenzoic acid)), 2-thiobarbituric acid (TBA), and 3-(4,5-dimethylthiazol-2-yl)-2,5-diphenyl tetrazolium bromide (MTT), as well as stains and chemicals used for histopathological studies, were procured from HiMEDIA Laboratories Pvt. Ltd., Mumbai, India.

### 2.2 Evaluation of cytotoxicity and nitrite in cell culture

The Raw 264.7 murine macrophage cell line, sourced from the National Center for Cell Science (NCCS) in Pune, India, was grown in DMEM medium supplemented with 10% fetal bovine serum (FBS) and 1% antibiotic solution (streptomycin–penicillin). The cultures were maintained in a sterile incubator at 37 °C with 5% CO_2_. Cytotoxicity of nootkatone was assessed using the 3-(4,5-dimethylthiazol-2-yl)-2,5-diphenyl tetrazolium bromide (MTT) assay according to established protocols. For nitrite estimation, RAW 264.7 cells were seeded in 96-well plates and allowed to adhere overnight. After adherence, the cells were pretreated with nootkatone (1.5 μM–50 μM) for 1 h and then stimulated with LPS (1 μg/mL) for 24 h. The culture supernatants were then collected for nitrite estimation and cytokine assays. Equal volumes of Griess reagent and cell culture supernatant were mixed and incubated in the dark for 10 min, after which absorbance was measured at a wavelength of 540 nm.

### 2.3 Estimating cytokines in cell culture

In a 96-well plate containing cultured Raw 264.7 macrophages, nootkatone was administered at concentrations ranging from 1.5 μM to 50 μM in the presence of LPS (1 μg/mL). Following a 24-h exposure period, the cell culture supernatants were collected to analyze TNF-*α* and IL-10 levels using ELISA kits, following the manufacturer’s instructions. Absorbance was measured at 450 nm and 570 nm, and the final cytokine concentrations (pg/mL) were calculated by subtracting the absorbance at 570 nm from that at 450 nm.

### 2.4 Experimental animals

Swiss albino mice, weighing 22–30 g and aged 6–8 weeks, were purchased from Jeeva Life Sciences, Hyderabad, India. The animals were kept in a 12-h dark/light cycle in a hygienic environment maintained at a temperature of 22–24 °C. They were acclimatized for 1 week prior to the start of the experiments. Throughout the study period, the animals were provided with a commercial standard rodent chow diet in the form of pellets and given purified water *ad libitum*. The experimental procedures were approved by the Institutional Animal Ethics Committee (Approval No.: 3/24/C.V.Sc., Hyd. IAEC-mice/12.06.2021).

### 2.5 Induction of lung injury

Before LPS instillation, the suspension was thoroughly mixed using a vortex shaker. Xylazine and ketamine were used to mildly anesthetize the animals, and rubber bands secured them on an inclined board. With blunt forceps, the mice’s tongues were gently pulled and held in place. A single dose of LPS (10 μg/kg) was then administered oropharyngeally to induce acute lung injury, as described in our earlier studies (8, 9).

### 2.6 Experimental design

A total of 30 Swiss albino male mice were used in the study. The animals were randomly divided into five groups (*n* = 6 per group) as follows:

Group I (Normal control, NC): Received normal saline intraperitoneally (i.p.) daily.

Group II (Disease control, DC): Induced with a single dose of LPS (10 μg/kg) via the oropharyngeal (o.p.) route on the 6th day of the experiment.

Group III (Nootkatone low dose, NLD): Pretreated with nootkatone (50 mg/kg, i.p.) for 7 days, then given LPS (10 μg/kg, o.p.) on the 6th day.

Group IV (Nootkatone high dose, NHD): Pretreated with nootkatone (100 mg/kg, i.p.) for 7 days, followed by LPS (10 μg/kg, o.p.) on the 6th day.

**Figure 1:**
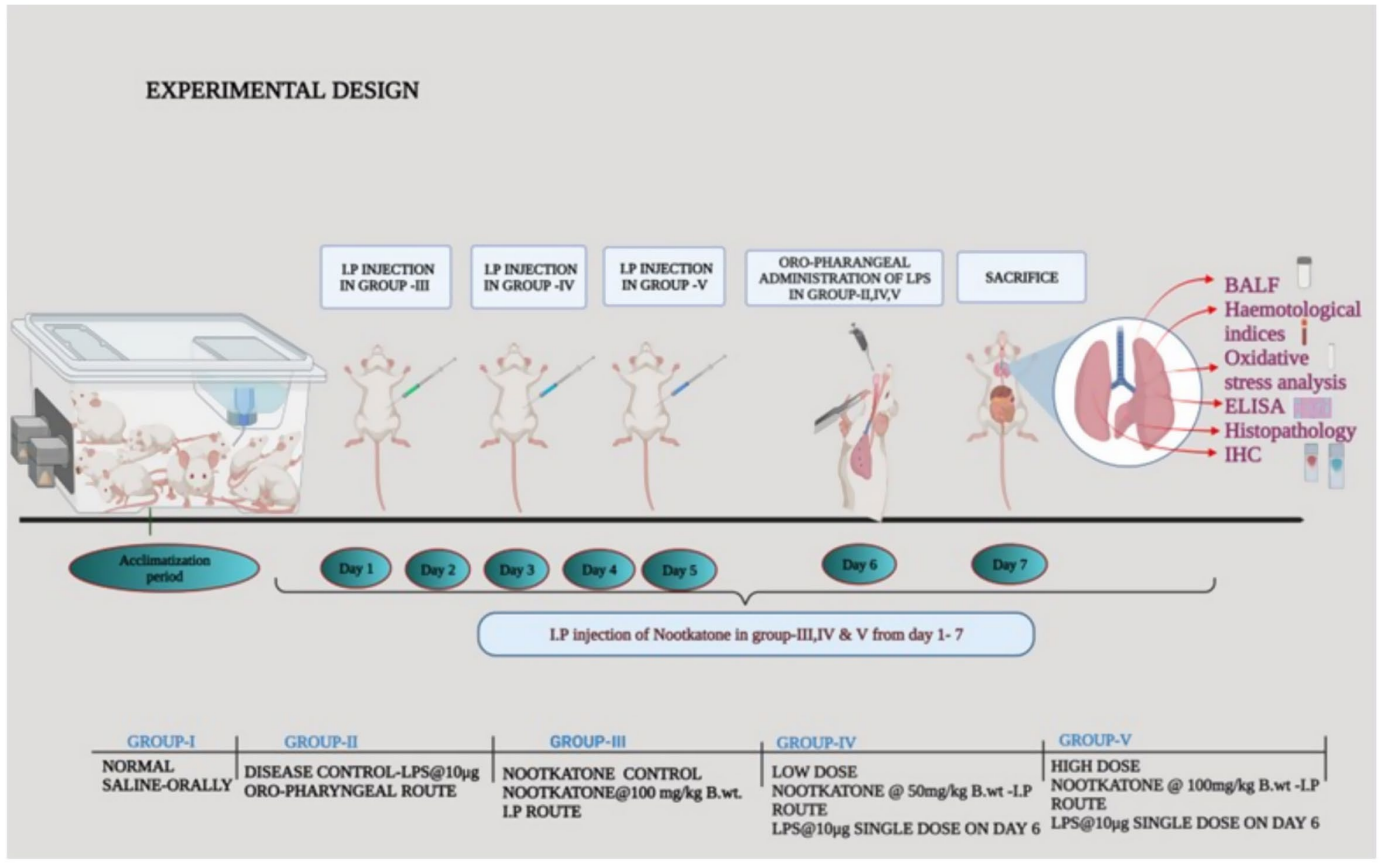
Experimental design illustrating the evaluation of nootkatone’s efficacy against LPS-induced acute lung injury.

Group V (Per se group, NPS): Pretreated with nootkatone (100 mg/kg, i.p.) for 7 days. On the 7th day, before euthanasia, blood was collected from all animals via the retro-orbital plexus using capillary tubes for hematological analysis (Humacount, Medsource Ozone Biochemicals Pvt. Ltd., India). At the end of the experiment (day 7), the animals were euthanized using compressed CO₂ delivered by the gradual-fill method (30–70% chamber volume/min). Following euthanasia, the lungs were collected, cleaned, and weighed for further analysis. Figure 1 depicts the experimental design of the study.

### 2.7 Bronchoalveolar lavage (BAL) fluid collection

For the collection of BAL fluid, 1 mL of phosphate-buffered saline (PBS) was instilled into the lungs and then aspirated using a catheter inserted into the trachea. The process was repeated three times to obtain sufficient lavage fluid for further analysis. An automated cell counter was used to determine differential cell counts in the BAL fluid (BALF) (10).

### 2.8 Measurement of oxidative and nitrosative stress parameters

Lipid peroxidation was evaluated by quantifying malondialdehyde (MDA), a marker of oxidative stress, using the thiobarbituric acid reactive substances (TBARS) assay. Briefly, 20 mg of lung tissue was homogenized in 500 μL of PBS. The homogenate was then mixed with SDS (8.1%), acetic acid (20%), thiobarbituric acid (0.8%), and distilled water. This mixture was heated in a water bath at 95 °C for 60 min and then cooled. After centrifugation at 10,000 rpm for 10 min, 200 μL of the supernatant was collected, and its absorbance was measured at a wavelength of 532 nm. The results were expressed as nM of MDA per mg of protein (11).

To measure glutathione, the method of Moron et al. was followed (12). Briefly, homogenized lung tissue was mixed with GSH buffer and Ellman’s reagent and incubated in the dark at room temperature for 10 min. The absorbance was then measured at 412 nm, and the results were expressed as μM/mg protein. Superoxide dismutase (SOD) enzyme activity was determined using a pyrogallol and MTT-based method. Absorbance was recorded at 570 nm, and enzyme activity was expressed as IU/mg protein (13). For estimating catalase activity, a hydrogen peroxide and dichromate-acetic acid solution–based method was used. The absorbance of the resulting green chromic acetate complex was measured at 610 nm, and enzyme activity was expressed as IU/mg protein (14). The levels of nitrite were measured using Griess reagent, with absorbance measured at 540 nm. The units of activity were expressed as μM/mg protein (15).

### 2.9 Cytokine profile

To quantify cytokine levels (IL-1β, IL-6, IL-10, IL-17, IL-22, TNF-*α*, IFN-*γ*, TGF-β1, and IL-10), an ELISA was conducted according to the manufacturer’s protocols. Briefly, 20 mg of lung tissue was homogenized in 1 mL of Tris–Triton lysis buffer containing protease inhibitors and then subjected to probe sonication (three cycles of 10s each). The homogenate was centrifuged at 10,000 rpm for 10 min at 4 °C, and the supernatant was collected and stored at −80 °C until further analysis. Cytokine levels were quantified following the protocol described in our earlier report (15). The data were expressed as pg. cytokine/mg protein (16).

### 2.10 Histopathological analysis

The lungs were preserved in 10% neutral buffered formalin (NBF) for histopathological examination. The tissues were processed through graded concentrations of alcohol, cleared in xylene, and embedded in paraffin at 55–56 °C. The paraffin blocks were sectioned into 5-μm-thick slices using a microtome. The sections were placed on grease-free glass slides pre-coated with Mayer’s egg albumin and incubated overnight at 37 °C for drying. The preserved tissues were then stained with hematoxylin and eosin (H&E). Histological injury was evaluated by two blinded examiners. The severity and extent of lung injuries were assessed using a 0–5 point scale across different categories, including congestion, emphysema, bronchiolar and alveolar damage, and infiltration of inflammatory cells.

### 2.11 Immunohistochemistry

For antigen retrieval, tissue sections were incubated with proteinase K (20 μg/mL) at 37 °C for 20 min after the deparaffinization and hydration process. Then, the sections were washed with TBS and treated with 3% H_2_O_2_ to inhibit endogenous peroxide activity. Afterward, they were blocked with 3% bovine serum albumin (BSA) for 1 h and incubated overnight at 4 °C with primary antibodies against Nrf2, NF-κB, COX-2, and TNF-*α* (1:200 dilution). The sections were then rinsed with TBS-T and incubated with a horseradish peroxidase-linked secondary antibody for 30 min, followed by additional washing in TBS-T. They were then stained with 3,3′-diaminobenzidine (DAB) and counterstained with hematoxylin the following day. After dehydration, the sections were mounted using DPX and examined under a microscope (Olympus Magcam – Magnus Microscope). Quantification of staining intensity was performed using ImageJ software (NIH) (17).

### 2.12 Analytical statistics

The experimental findings are expressed as mean ± standard error (SE). Statistical analysis was conducted using GraphPad Prism version 5.0 software. One-way analysis of variance (ANOVA), followed by Tukey’s multiple comparison test, was applied to evaluate differences among the groups. A *p*-value of <0.05 was considered statistically significant.

## 3 Results

### 3.1 Nootkatone effects on cytotoxicity, nitrite, IL-10, and TNF-*α* levels in an *in vitro* cell culture study

**Figure 2:**
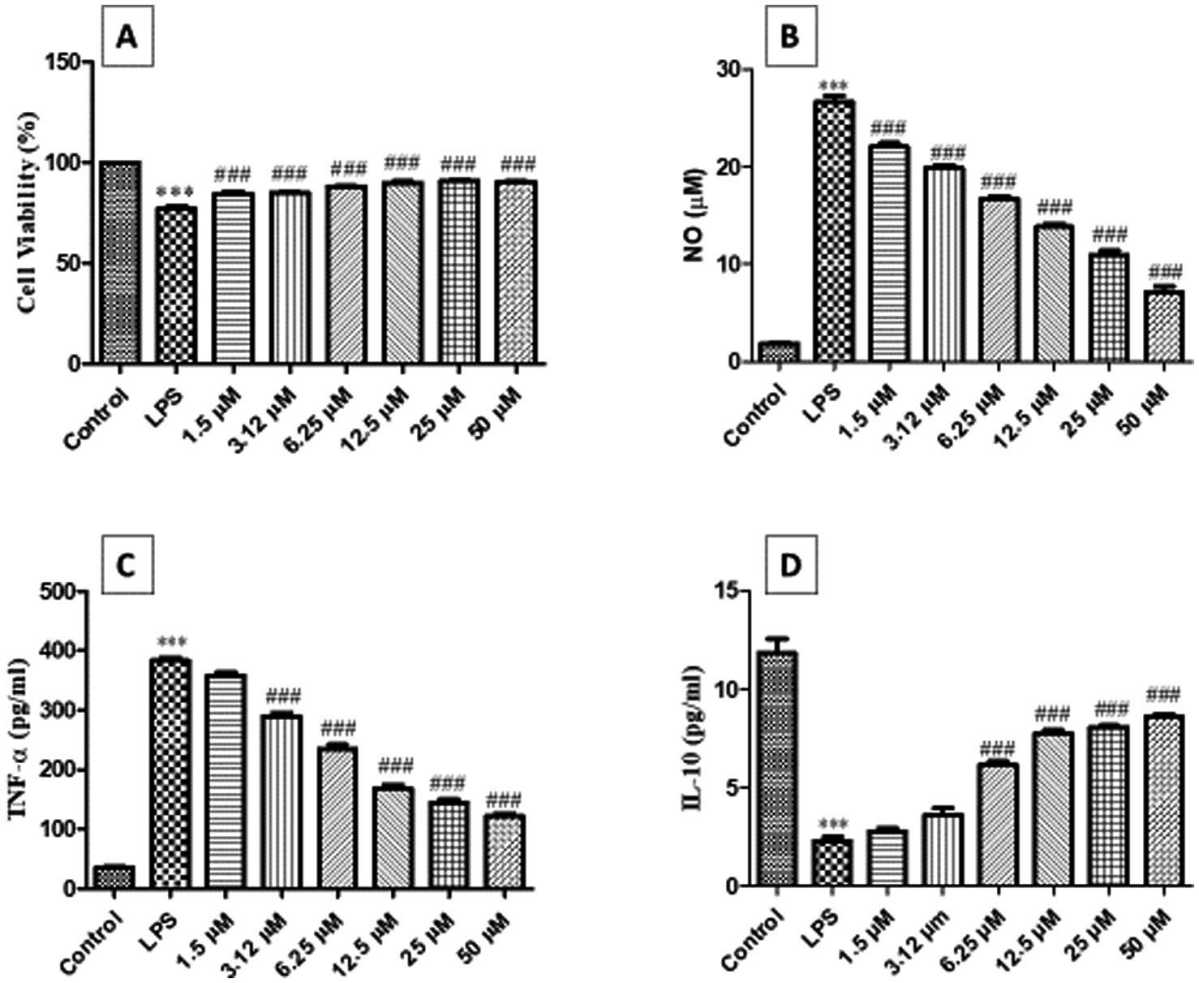
The effect of nootkatone on cell viability (%) **(A)**, nitrite levels **(B)**, TNF-*α* **(C)**, and IL-10 **(D)** was assessed in RAW 264.7 cells exposed to LPS (1 μg/mL) with or without nootkatone treatment. The results are presented as mean ± SEM (*n* = 6). Statistical significance: ***p < 0.001 vs. negative control (NC); ^#^*p* < 0.05, ^##^*p* < 0.01, and ^###^*p* < 0.001 vs. disease control (DC). Statistical analysis was conducted using one-way ANOVA followed by Tukey’s multiple comparisons test.

To assess the anti-inflammatory activity of nootkatone, we conducted an *in vitro* cell culture study using RAW 264.7 macrophage cells. The results showed that graded concentrations of nootkatone (1.5 μM to 50 μM) demonstrated a favorable safety profile, with a lower percentage of cell death, indicating minimal cytotoxicity (Figure 2A), along with a significant reduction in nitrosative stress (Figure 2B). Additionally, the assessment of inflammatory cytokines revealed a significant, concentration-dependent decrease in TNF-*α* levels, accompanied by a marked increase in the anti-inflammatory cytokine IL-10 (*p* < 0.001) compared to LPS-treated cells (Figures 2C,D). These findings collectively highlight the potent anti-inflammatory properties of nootkatone in LPS-stimulated macrophages.

### 3.2 Effect of nootkatone on LPS-treated lung indices

**Figure 3:**
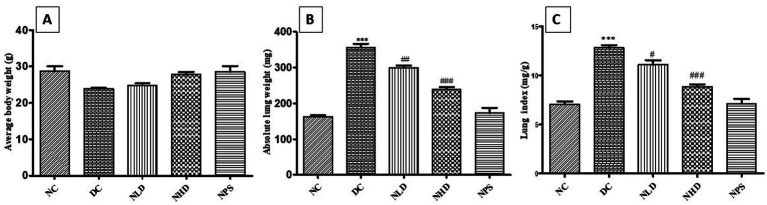
The effect of nootkatone on the lung index. **(A)** Average body weight: We found that the DC group animals did not show a significant reduction in body weight compared to the NC group. Treatment with nootkatone resulted in a considerable increase in body weight. **(B)** Absolute lung weight: We found that the DC group animals exhibited a significant increase in lung weight compared to the NC group, while nootkatone treatment led to a considerable reduction. **(C)** Lung index: The lung index in the DC group increased significantly compared to the NC group, whereas nootkatone treatment produced a substantial reduction in the lung-to-body weight ratio. Data are expressed as mean ± SEM (*n* = 6). ***p < 0.001 vs. NC; ^#^*p* < 0.05, ^##^ *p* < 0.01, and ^###^*p* < 0.001 vs. DC (Tukey’s multiple comparisons test was used following one-way ANOVA).

The effects of nootkatone were evaluated in a lung-damage model induced by oropharyngeal LPS administration. Mice in the disease control (Group II) showed a significant reduction in body weight compared to the normal control (Group I). In contrast, the groups treated with nootkatone (NLD and NHD) demonstrated noticeable improvement in body weight relative to the LPS-treated group (Figure 3A). The average absolute lung weights (mg) of the LPS-treated group were substantially increased (*p* < 0.001) compared with Group I. However, both treatment groups, NLD and NHD, exhibited a dose-dependent reduction in lung weight (*p* < 0.01 and *p* <  0.001, respectively, Figure 3B). Pulmonary edema is the pathognomonic lesion of ALI, which is characterized by an increased lung index (relative lung weight). The index of the lungs in the DC group was observed to be significantly higher (*p* < 0.001) than that of the NC group, showing that LPS-based induction of lung injury was successful (Figure 3C). In contrast, compared to the DC group, the lung index of the treatment groups was statistically decreased at both doses (*p* < 0.05 in NLD and *p* < 0.001 in NHD, Figure 3C). There were no alterations in the NPS groups, indicating the safety of the compound.

### 3.3 Effect of nootkatone on LPS-induced changes in BALF parameters

**Figure 4:**
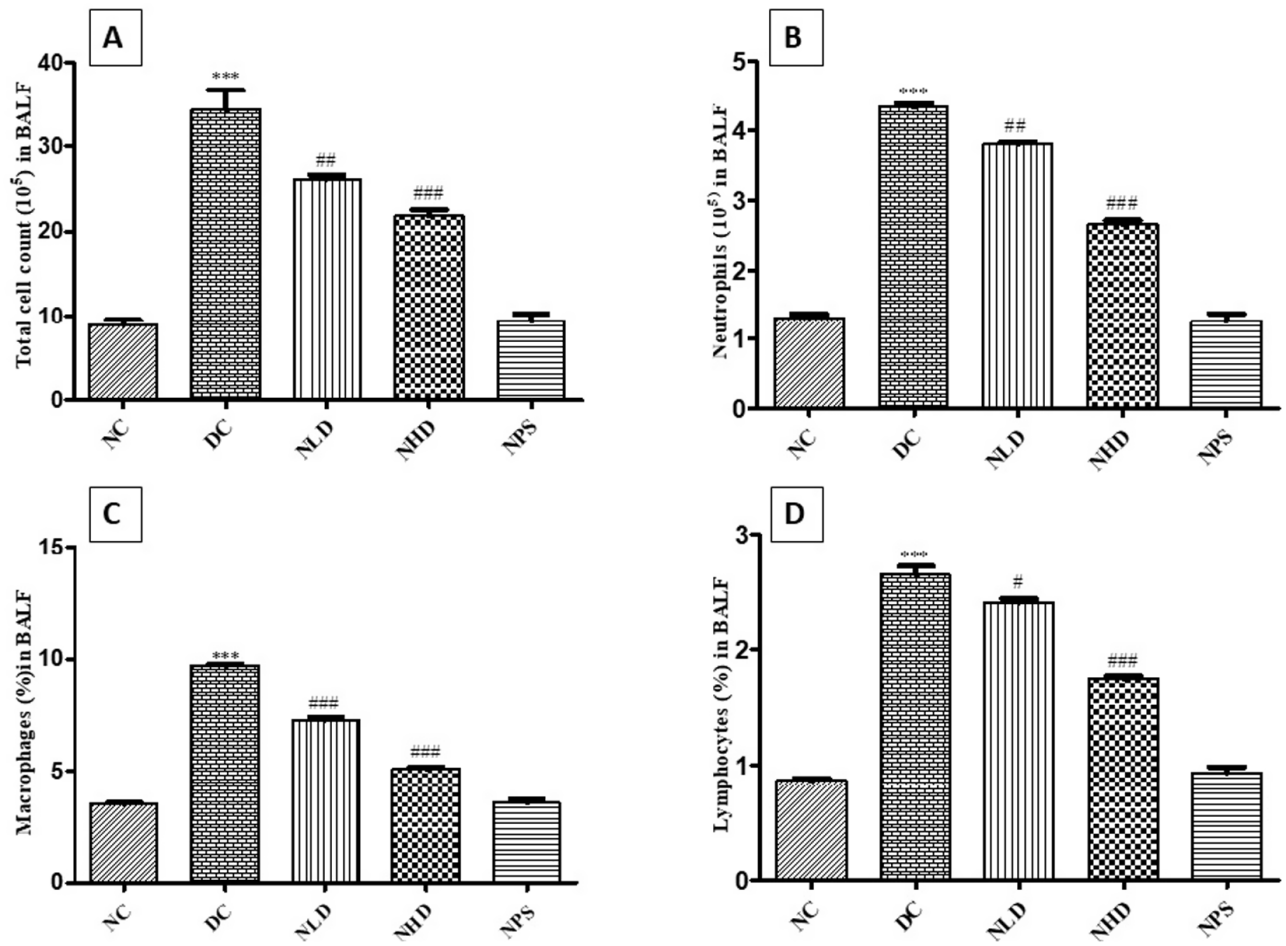
Effect of nootkatone on BALF parameters — total cell count (TCC) **(A)**, neutrophils **(B)**, macrophages **(C)**, and lymphocytes **(D)**. Compared to the NC group, various soluble and cytological BALF characteristics were significantly elevated in the DC group. Treatment with nootkatone resulted in a substantial reduction in these markers. Data are presented as mean ± SEM (*n* = 6). *** *p* < 0.001 vs. NC; ^#^*p* < 0.05, ^##^*p* < 0.01, and ^###^*p* < 0.001 vs. DC (Tukey’s multiple comparisons test was used following one-way ANOVA).

The study evaluated the inflammatory cell counts in bronchoalveolar lavage fluid (BALF) following LPS treatment and examined the effect of nootkatone. Total cell, lymphocyte, neutrophil, and macrophage counts were significantly higher (*p* < 0.001, Figures 4A–D) in LPS-treated mice compared to the NC group. In comparison to the LPS-treated group, nootkatone administration resulted in significant reductions in total cell counts (*p* < 0.01 in NLD; *p* < 0.001 in NHD, Figure 4A), neutrophil counts (*p* < 0.01 in NLD; *p* < 0.001 in NHD, Figure 4B), macrophage counts (*p* < 0.001 at both doses; Figure 4C), and lymphocyte counts (*p* < 0.05 in NLD; *p* < 0.001 in NHD; Figure 4D). These findings indicate that nootkatone mitigates LPS-induced lung injury by reducing inflammatory cell infiltration.

### 3.4 Effect of nootkatone on LPS-induced changes in blood parameters

**Figure 5:**
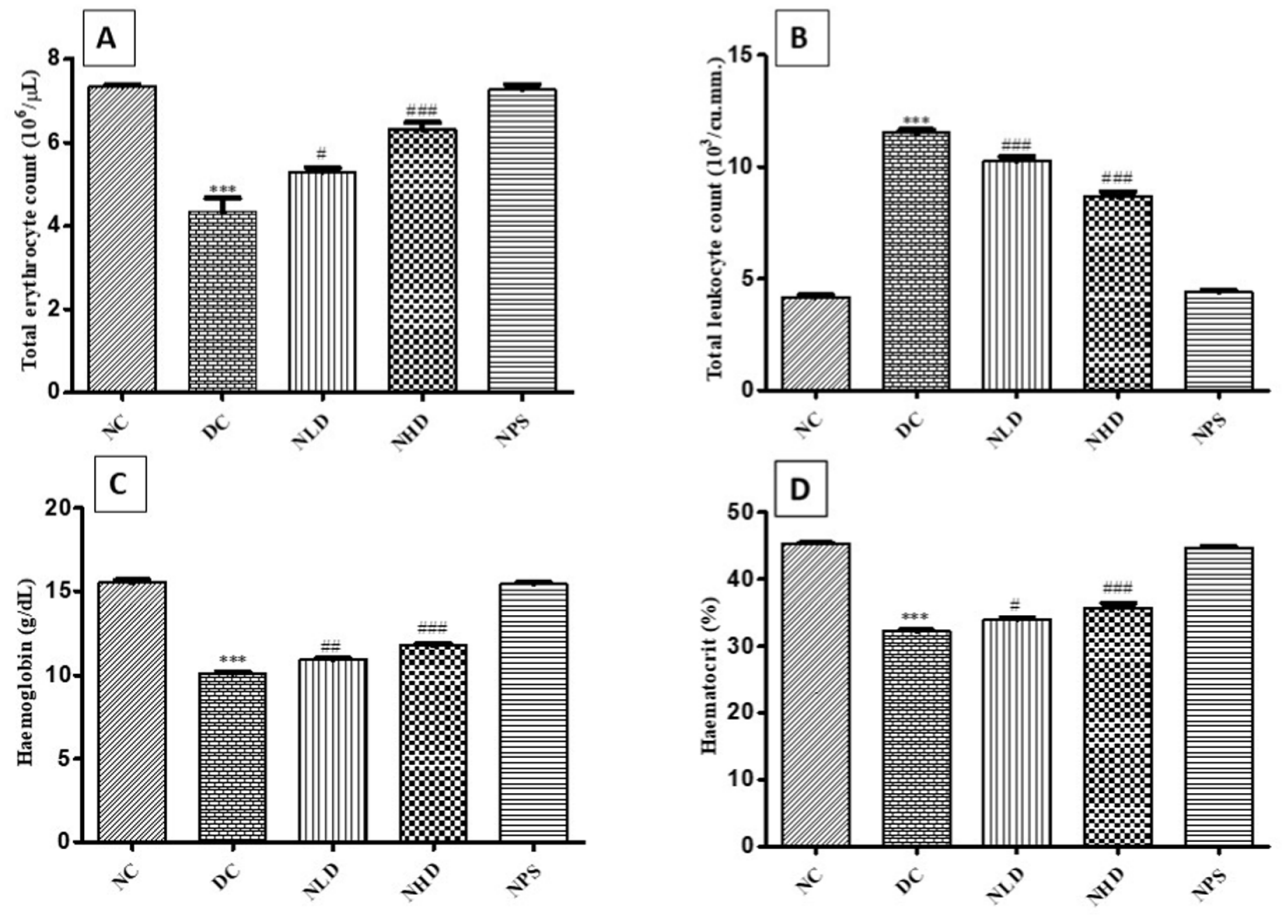
Effect of nootkatone on hematological parameters — **(A)** TEC, **(B)** TLC, **(C)** hemoglobin, and **(D)** hematocrit. The hematological parameters showed significant alterations in the DC group compared to the NC group. Treatment with nootkatone resulted in notably higher hematological values. Data are shown as mean ± SEM (*n* = 6). ***p < 0.001 vs. NC; ^#^ *p* < 0.05, ^##^ *p* < 0.01, and ^###^ *p* < 0.001 vs. DC (Tukey’s multiple comparisons test was used following one-way ANOVA).

The blood indices, including total erythrocyte count (TEC), total leukocyte count (TLC), hemoglobin (Hb), and hematocrit (HCT), were significantly lower (*p* < 0.001, Figures 5A–D) in LPS-treated mice compared to the normal control group. However, the administration of nootkatone resulted in a marked improvement in these parameters. TEC showed a significant increase (*p* < 0.05 in NLD and *p* < 0.001 in NHD; Figure 5A), while TLC also improved significantly (*p* < 0.001 at both doses; Figure 5B). Similarly, Hb levels (*p* < 0.01 in NLD and *p* < 0.001 in NHD) and hematocrit values (*p* < 0.05 in NLD and *p* < 0.001 in NHD) were significantly elevated compared to the LPS-treated group. These results indicate that nootkatone exerted a dose-dependent hemoprotective effect against LPS-induced alterations in blood parameters (Figures 5C,D).

### 3.5 Effect of nootkatone on oxidative stress parameters in LPS-mediated lung injury

**Figure 6:**
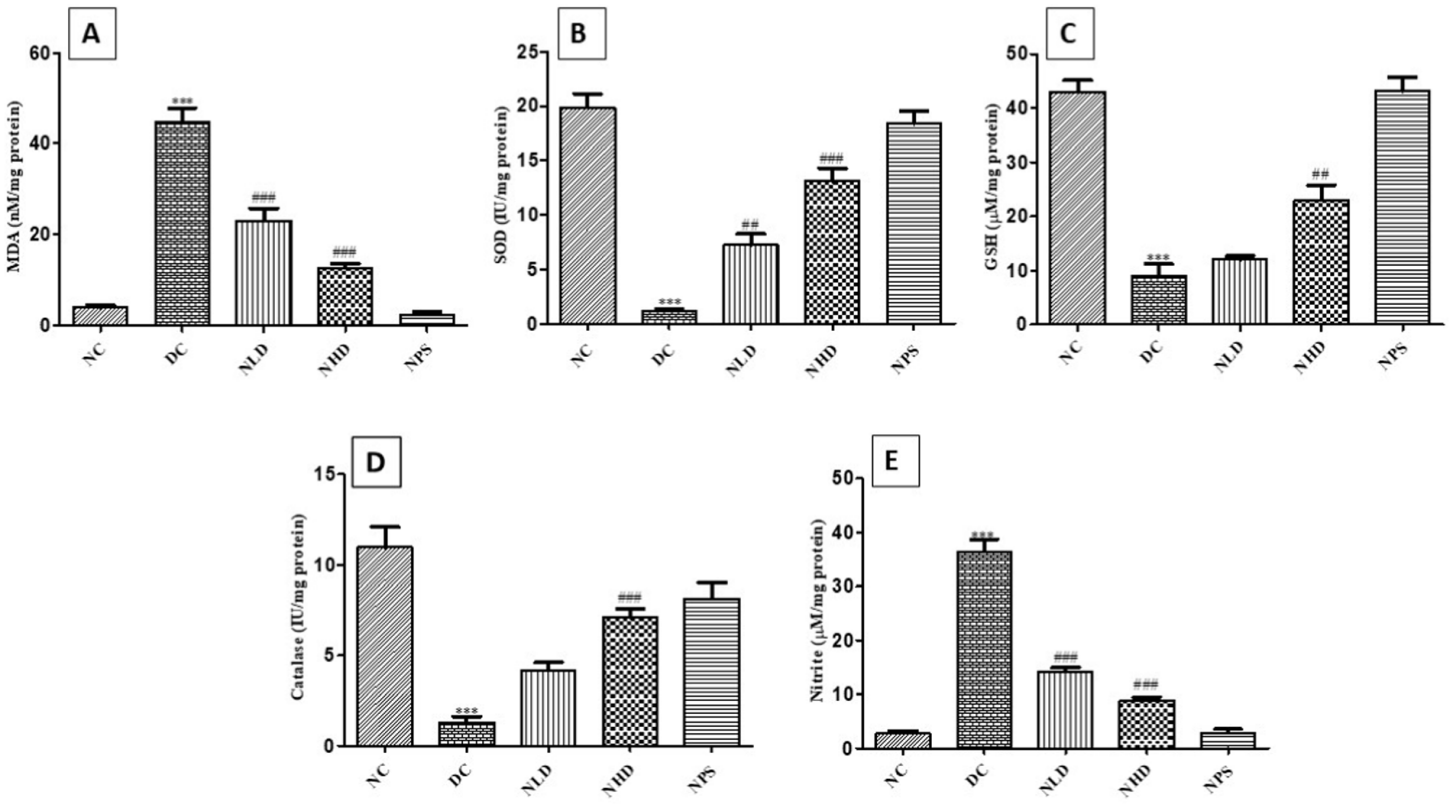
Effect of nootkatone on LPS-induced oxidative and nitrosative stress. Concentrations of **(A)** MDA, **(B)** SOD, **(C)** GSH, **(D)** catalase, and **(E)** nitrite are shown. MDA and nitric oxide levels in the DC group remained substantially higher than those in the NC group, whereas nootkatone treatment resulted in these levels. Conversely, GSH, SOD, and catalase levels were considerably lower in the DC group than in the NC group; treatment with nootkatone significantly increased their levels compared to the DC group. Data are shown as mean ± SEM (*n* = 6). ***p < 0.001 vs. NC; ^##^*p* < 0.01, and ^###^*p* < 0.001 vs. DC (Tukey’s multiple comparisons test was used following one-way ANOVA).

The concentration of malondialdehyde (MDA) was significantly higher (*p* < 0.001) in the LPS-treated group compared to Group I, indicating elevated lipid peroxidation. Compared to the DC group, treatment with nootkatone led to a marked reduction in MDA levels (*p* < 0.001 at both doses; Figure 6A).

Superoxide dismutase (SOD), glutathione (GSH), and catalase activities were significantly lower (*p* < 0.001) in LPS-treated mice compared to the NC group, indicating oxidative stress. However, nootkatone treatment notably restored these antioxidant enzyme activities. Specifically, SOD levels increased significantly (*p* < 0.01 in NLD and *p* < 0.001 in NHD; Figure 6B), GSH concentrations rose significantly (*p* < 0.01 in NHD; Figure 6C), and catalase activity showed a pronounced increase (*p* < 0.001 in NHD; Figure 6D) compared to the LPS-treated mice. Furthermore, in the DC group, GSH concentrations were substantially lower (*p* < 0.001) than in the NC group. Compared to the DC group, nootkatone treatment led to a substantial rise (*p* < 0.01 in NHD) in GSH concentrations (Figure 6C). The DC group also showed considerably reduced catalase activity (*p* < 0.001) compared to the NC group. In comparison to the DC group, nootkatone administration resulted in a substantial increase (*p* < 0.001 in NHD; Figure 6D) in catalase activity. Moreover, tissue nitrite concentrations in the DC group revealed a substantial increase (*p* < 0.001) compared to the NC group, whereas both the NLD and NHD groups exhibited a significant decrease compared to the DC group (*p* < 0.001 at both doses; Figure 6E). In the NPS group, the levels of these biological markers were normal, suggesting that the compound is safe. These findings indicate that the antioxidant and anti-nitrosative stress properties of nootkatone may be responsible for the observed protective effects.

### 3.6 Effect of nootkatone on inflammatory signaling pathways in LPS-treated mice

**Figure 7:**
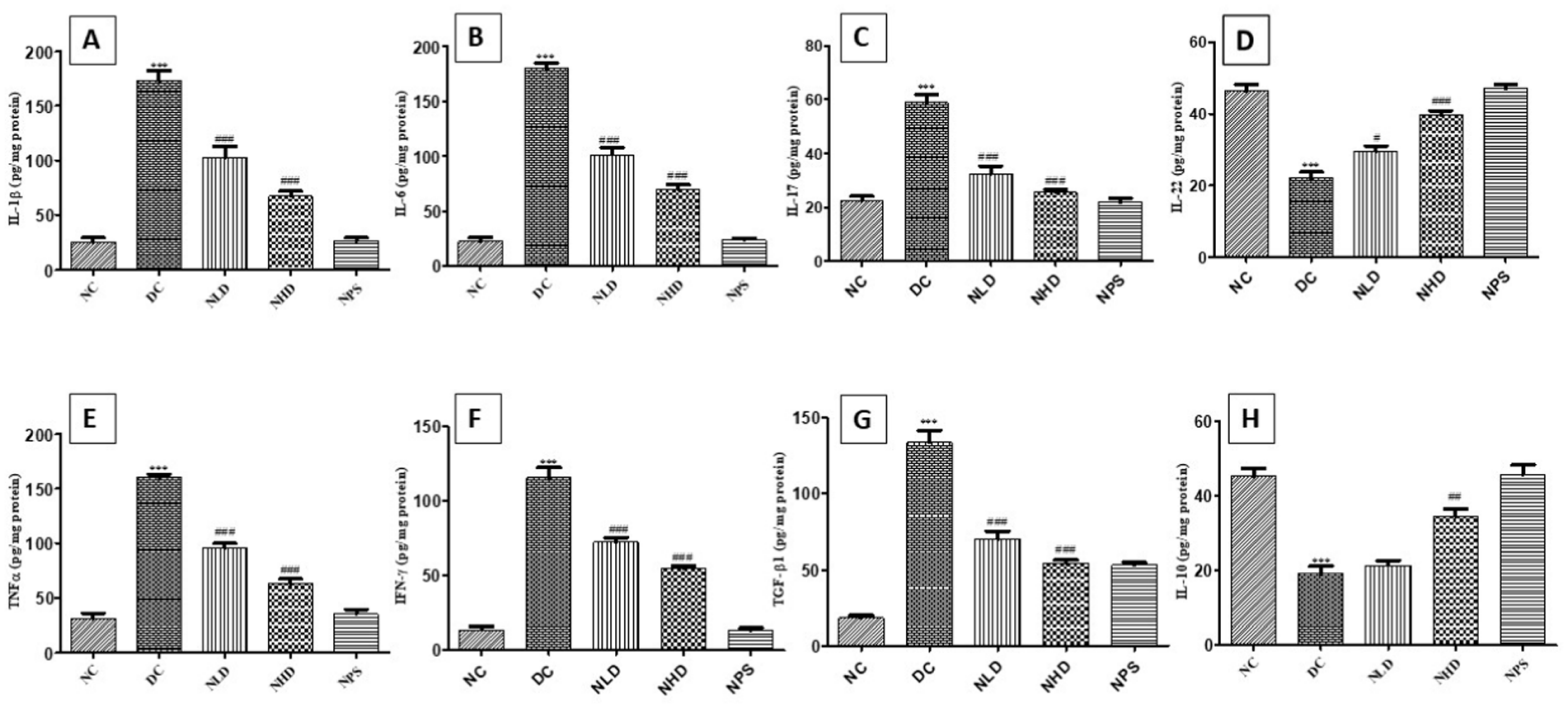
Effect of nootkatone on pro-inflammatory cytokines. **(A)** IL-1β, **(B)** IL-6, **(C)** IL-17, **(D)** IL-22, **(E)** TNF-α, **(F)** IFN-γ, **(G)** TGF-β, **(H)**, and IL-10. Except for IL-10, cytokine levels were significantly lower in the nootkatone-treated groups compared to the DC group. Data are shown as mean ± SEM (*n* = 6). ***p < 0.001 vs. NC; ^#^*p* < 0.05, ^##^ *p* < 0.01, and ^###^*p* < 0.001 vs. DC (Tukey’s multiple comparisons test was used following one-way ANOVA).

In this study, we explored inflammatory markers and anti-inflammatory markers using ELISA. The cytokine levels were found to be elevated in the LPS-treated group of animals. In comparison to the NC group, LPS administration resulted in substantially higher levels (*p* < 0.001) of IL-1β, IL-6, IL-17, IL-22, TNF-*α*, IFN-*γ*, and TGF-β1 (Figure 7). In contrast, nootkatone treatment produced significant improvement over the LPS-treated group. The reductions in inflammatory cytokines were as follows: IL-1β (*p* < 0.001 in NLD and NHD; Figure 7A), IL-6 (*p* < 0.001 in NLD and NHD; Figure 7B), IL-17 (*p* < 0.001 in NLD and NHD; Figure 7C), IL-22 (*p* < 0.05 in NLD and *p* < 0.001 in NHD; Figure 7D), TNF-α (*p* < 0.001 in both NLD and NHD; Figure 7E), IFN-γ (*p* < 0.001 in both NLD and NHD; Figure 7F), and TGF-β1 (*p* < 0.001 in both NLD and NHD; Figure 7G). Additionally, the anti-inflammatory cytokine IL-10 was significantly lower (*p* < 0.001; Figure 7H) in the LPS-treated group compared to the NC group. Conversely, the treatment groups NLD and NHD showed a marked improvement, with a particularly pronounced effect observed in the NHD group (Figure 7H), compared to the LPS-treated mice. We found that there were no abnormal changes in cytokine levels in the NPS group, indicating that this novel phytoconstituent is safe. These findings clearly show that nootkatone has strong anti-inflammatory properties.

### 3.7 Effect of nootkatone on the morphological and histopathological changes in the lungs of LPS-treated mice

**Figure 8:**
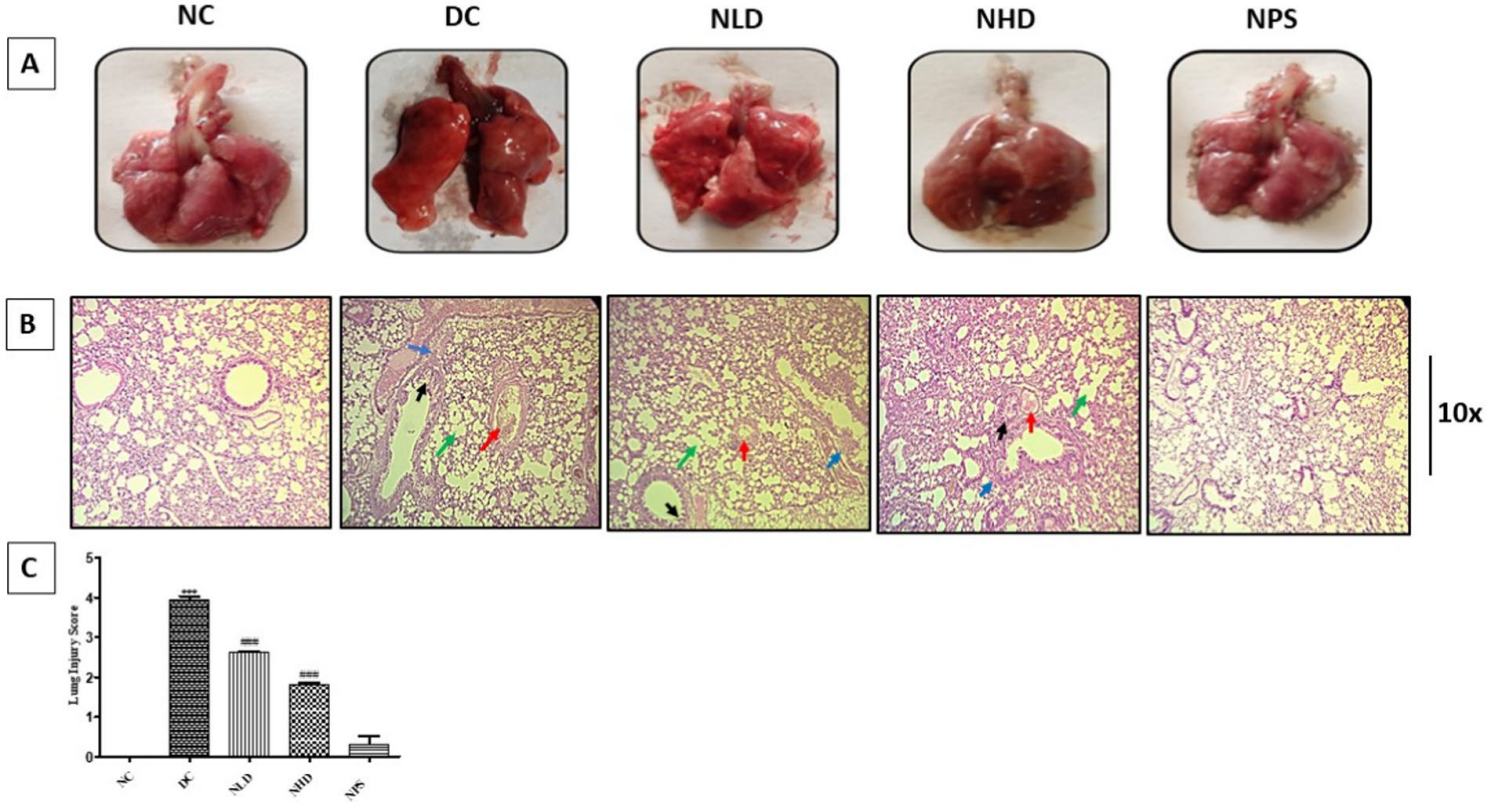
Effect of nootkatone on lung morphology **(A)**, histology **(B)**, and lung injury score **(C)**. Data are presented as mean ± SEM (*n* = 6). *** *p* < 0.001 vs. NC; ^###^*p* < 0.001 vs. DC (Tukey’s multiple comparisons test was used following one-way ANOVA). Histological images show congestion (red arrow), emphysema (green arrow), damage to bronchioles and alveoli (black arrow), and the infiltration of inflammatory cells (blue arrow).

In the LPS-treated group, we observed distinct abnormalities in lung morphology, including severe congestion and edema, compared to the normal control group. In contrast, the lungs of nootkatone-treated mice showed noticeably less damage than those of the LPS-only treated group. Nootkatone administered alone in the NPS group did not cause any abnormal morphological responses, confirming the safety of the compound (Figure 8A). Histopathological examination of lung tissue sections from the NC group revealed normal histoarchitecture, characterized by numerous intact alveoli, bronchioles lined with normal epithelium, thin alveolar septa, and normally structured venules.

In contrast, the DC group exhibited severely disrupted lung architecture, characterized by significant congestion and emphysema, collapsed alveolar walls, accumulation of edematous fluid within the alveolar lumen, constricted bronchioles, desquamated epithelial cells within the alveolar spaces, peribronchiolar edema, mild hyperplasia, and infiltration of inflammatory cells. The NLD group exhibited moderate histological alterations, including partial disruption of lung structure, moderate thickening of alveolar septa, moderate changes in bronchial and alveolar lumens, emphysema, and infiltration of inflammatory cells. In the NHD group, lung sections showed evidence of alveolar epithelial regeneration, typical bronchial epithelium, normal venule morphology, mild congestion, minimal emphysema, and limited inflammatory cell infiltration. The histoarchitecture of lung sections from the NPS group was identical to that of the NC group, displaying a normal and intact structure (Figure 8B).

### 3.8 Effect of nootkatone on the lung injury score in LPS-treated mice

To evaluate lung injury across different treatment groups, the assessment included the extent of inflammatory cell infiltration within the bronchial and alveolar lumina, the accumulation of edematous fluid in and around these structures, the thickening of inter-alveolar septa, and the presence of hemorrhages in different lobes, as well as signs of emphysema. A 0–5 point scale was used to grade the severity and degree of lung injury, where scores of 4–5 indicated severe lung injury, 2–4 indicated moderate injury, and scores below 2–1 indicated mild injury. The lung injury score in LPS-treated animals was significantly higher (*p* < 0.001; Figure 8C) compared to the untreated Group I, while the treatment groups NLD and NHD showed mild and moderate lung injury, respectively. Furthermore, the histological characteristics and scores of the NPS group were similar to those of the NC group, demonstrating that our intervention had no harmful consequences (Figure 8C).

### 3.9 Effect of nootkatone on anti-inflammatory and antioxidant activity in LPS-treated mice as assessed using immunohistochemistry

**Figure 9:**
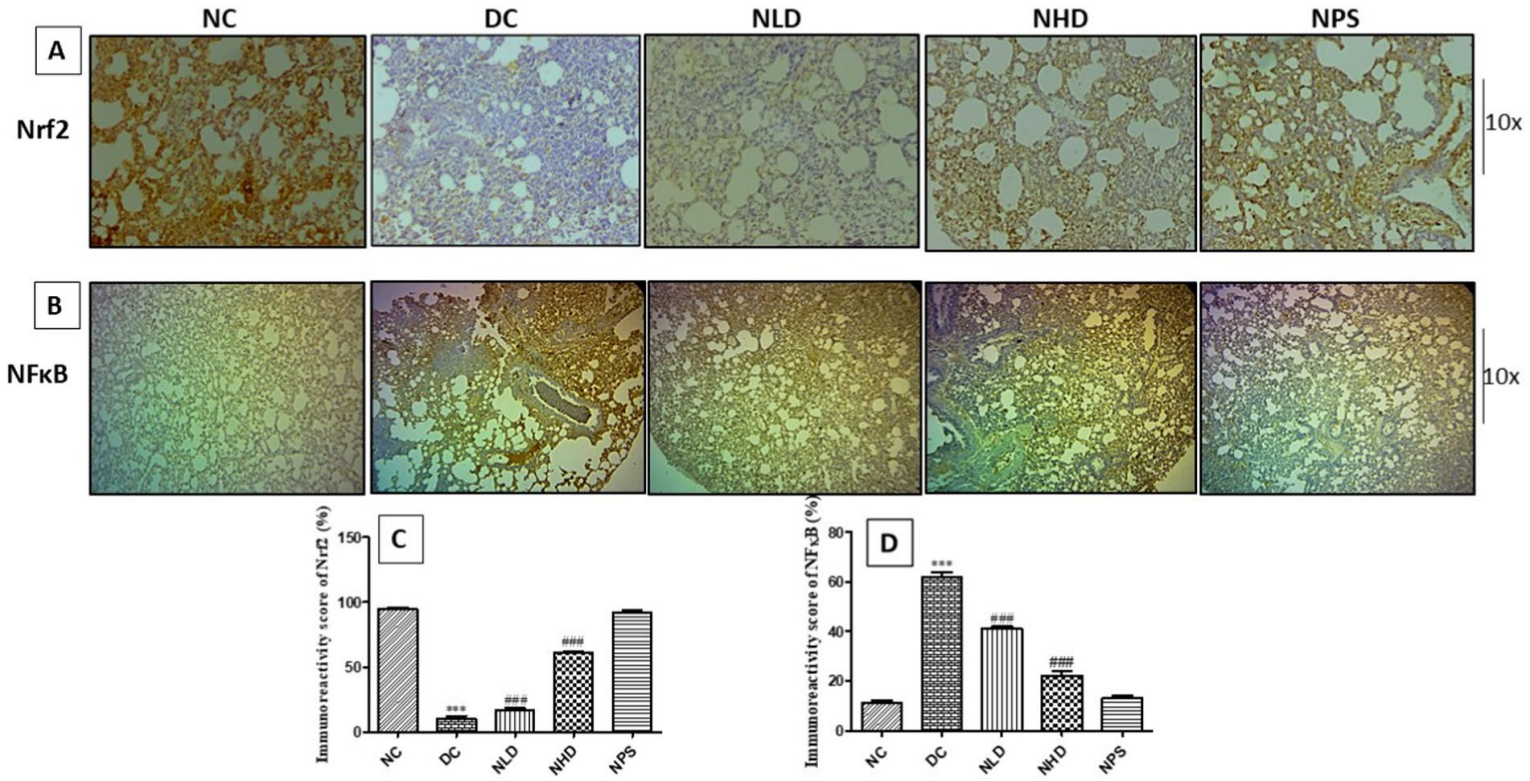
Effect of nootkatone on the immunohistochemical expression of Nrf-2 **(A)** and NF-κb **(B)**; **(C,D)** Quantitative immunoreactivity scores (mean ± SEM) (*n* = 6). ***p < 0.001 vs. NC; ^###^*p* < 0.001 vs. DC (Tukey’s multiple comparisons test was used following one-way ANOVA).

**Figure 10:**
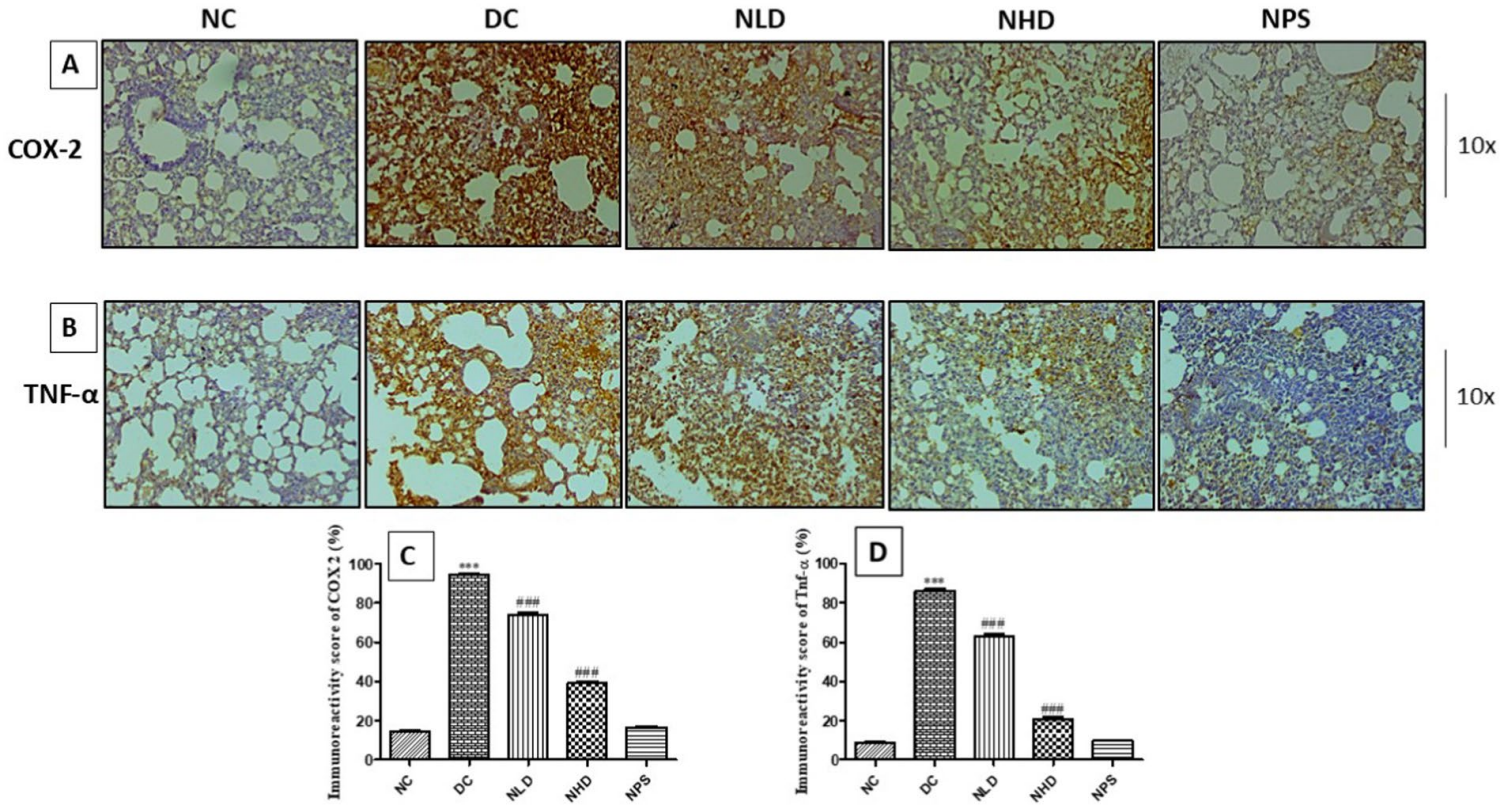
Effect of nootkatone on the immunohistochemical expression of COX-2 **(A)** and TNF-α **(B)**; **(C,D)** Quantitative immunoreactivity scores (mean ± SEM) (*n* = 6). ***p < 0.001 vs. NC; ^###^*p* < 0.001 vs. DC (Tukey’s multiple comparisons test was used following one-way ANOVA).

The study was conducted to evaluate the anti-inflammatory and antioxidant properties of nootkatone in LPS-treated mice. Immunostaining for Nrf-2, an antioxidant nuclear transcription factor, showed a significant reduction in expression in the LPS-treated group (*p* < 0.001, Figures 9A,C) compared to the NC group. In contrast, the treatment groups NLD, NHD, and NPS showed intense positive immunoreactivity for the Nrf-2 protein. The increased Nrf-2 expression in the lung tissues of these groups indicates an enhanced antioxidant response regulated by nootkatone (Figures 9A,C). Immunohistochemical analysis of lung sections from the LPS-treated group revealed strong positive staining for NF-κB (*p* < 0.001, Figures 9B,D), COX-2 (*p* < 0.001, Figures 10A,C), and TNF-*α* (*p* < 0.001, Figures 10B,D), indicated by intense brown coloration compared to the untreated control sections. In contrast, the NPS group showed only mild immunostaining, while the NLD and NHD treatment groups showed moderate to mild immunostaining for NF-κB protein (*p* < 0.001 at both doses; Figures 9B,D), COX-2 (*p* < 0.001 at both doses; Figures 10A,C), and TNF-α (*p* < 0.001 at both doses; Figures 10B,D). These results demonstrate that nootkatone treatment at both doses exerts significant anti-inflammatory effects in a dose-dependent manner likely through the modulation of NF-κB, COX-2, and TNF-α signaling pathways, along with the upregulation of Nrf-2-mediated antioxidant defense.

## 4 Discussion

The rupture of the alveolar–capillary membrane leads to ALI, a critical experimental condition associated with high morbidity and mortality rates (18). The development of ALI is characterized by epithelial barrier breakdown, neutrophil infiltration, pulmonary edema, severe hypoxia, and acute pulmonary inflammatory responses in the lungs (19). The inoculation of lipopolysaccharide is frequently used preclinically to develop a lung damage model for investigating the pathophysiology of ALI and screening new pharmacological inhibitors. Acute exposure to LPS activates the innate immune system, causing a surge in inflammatory cells, a cytokine storm, and increased capillary permeability in the lungs, all of which contribute to pulmonary edema (20).

The pathogenesis of apoptotic alterations in lung tissue during ALI is believed to result from delayed and abnormal production of neutrophil granulocytes, along with dysregulated cytokine release. Infiltrated neutrophils, which play a critical role in neutrophil-mediated ALI damage and disease progression, produce reactive oxygen species (ROS), leading to a redox imbalance (21). Furthermore, macrophages and neutrophils release pro-inflammatory cytokines that, in the later stages of ALI, act as key mediators of the inflammatory response, modulating fibroblast activity and contributing to oxidative stress (22, 23). During the acute inflammation process, there is a sharp increase in inflammatory cytokines, which is a key characteristic of LPS-induced ALI (24).

Nootkatone, a sesquiterpenoid compound, is a highly valued aromatic molecule naturally found in grapefruit, pummelo, and the Nootka cypress tree. Recent reports have highlighted the intriguing therapeutic potential of nootkatone and its metabolites, demonstrating a wide range of biological activities (25). Given the limited success of current therapeutic interventions for ALI/ARDS, further research into novel and effective medicinal approaches is urgently required (26). In the present study, we evaluated the anti-inflammatory potential of nootkatone, a potent phytochemical with promising pharmacological properties, through both *in vitro* and *in vivo* approaches. The *in vitro* investigations involved the use of murine macrophages (Raw 264.7) stimulated with LPS, while the *in vivo* studies were conducted using two distinct doses of nootkatone tested against LPS-induced acute lung injury (ALI). To date, no scientific literature has reported the effects of nootkatone on LPS-induced ALI. Therefore, the present study aims to address this gap. Pharmacokinetic parameters such as plasma half-life and volume of distribution for nootkatone have not been clearly reported in the current literature, including the review by Jha et al. (27), indicating the need for further investigation. *In vitro* examination of nootkatone’s protective effects demonstrated a significant decrease in nitrite release from LPS-stimulated macrophages, accompanied by enhanced cell viability. Additionally, nootkatone notably reduced TNF-*α* levels while elevating IL-10 levels in the cellular supernatant, indicating its beneficial anti-inflammatory effects (28).

Previous experimental studies have demonstrated that during the acute inflammation phase, there is a pronounced increase in inflammatory cytokines, a key characteristic of LPS-induced ALI. Reactive oxygen species (ROS), generated by infiltrating neutrophils—which play a crucial role in the pathogenesis of neutrophil-mediated ALI—lead to an imbalance between oxidant and antioxidant factors (24). This process is primarily mediated through the activation of the NF-κB and MAPK pathways, leading to alveolar epithelial damage, loss of surfactant function, and impaired lung physiology. The successful induction of ALI is characterized by an increase in absolute and relative lung weights due to the infiltration of polymorphonuclear cells and the accumulation of non-cardiogenic proteinaceous fluid.

Additionally, elevated oxidative (TBARS) and nitrosative (NO) stress, along with a significant reduction in antioxidant enzymes (GSH, SOD, and CAT), was observed. This was accompanied by a surge in pro-inflammatory cytokines (IL-6, IL-1β, IL-17, IL-22, IFN-*γ*, TGF-β1, and TNF-α) and a marked decline in the anti-inflammatory cytokine IL-10 (29, 30). We found a non-significant reduction in the body weights of DC mice compared to those of the NC group, which may be attributed to the sharp rise in pro-inflammatory cytokines and increased oxidative stress, leading to rapid catabolism and lethargy. These findings are consistent with those of prior research (1, 31). Weight gain in the NLD and NHD groups was observed and was comparable to the mean body weights of mice in the NC and NPS groups. This beneficial impact may be attributed to the anti-inflammatory and antioxidant properties of nootkatone.

Lung edema, a pathognomonic lesion of LPS-induced ALI, is characterized by significantly elevated absolute and relative lung weights, which could be due to increased oxidative stress, elevated inflammatory cytokines, and capillary injury. These changes promote the leakage of proteins and other cellular components into the pulmonary interstitium (32). In our study, we observed a significant increase in both absolute and relative lung weights in the DC group compared to the NC and NPS groups. This significant increase could be due to a severe inflammatory response, which is consistent with previous findings (1, 31, 32). In contrast, the nootkatone-treated groups (NLD and NHD) showed significant improvement, reflected by a reduction in the lung index. This reduction might be due to the restoration of the antioxidant defense system, decreased free radical–induced oxidative damage, and the anti-inflammatory effects of nootkatone.

Activated alveolar macrophages and neutrophils contribute to excessive nitric oxide (NO) production through the expression of inducible nitric oxide synthase (iNOS), resulting in damage to lung tissues. Additionally, secretory cells, monocytes, and macrophages form a complex cytokine network that initiates, amplifies, and sustains the inflammatory response in ALI/ARDS. Bronchoalveolar lavage fluid (BALF) serves as a valuable matrix for assessing the extent of ALI induced by various pathogens, including LPS (33). In the BALF of the DC group, there was an increase in total cell count (TCC), neutrophils, macrophages, and lymphocytes, which indicates the development of pulmonary edema. In our study, the total cell count, neutrophils, macrophages, and lymphocytes were substantially higher in the DC group compared to the NC group, confirming the successful induction of LPS-mediated lung inflammation.

A significant increase in inflammatory cell infiltration promotes an exaggerated activation of the host immune response, leading to excessive cytokine release from activated neutrophils. In our study, nootkatone treatment resulted in a decrease in TCC, neutrophils, macrophages, and lymphocytes compared to the DC group. The ameliorative effect of nootkatone may be attributed to its anti-inflammatory and antioxidant properties, indicating its protective role in reducing the LPS-induced increase in pulmonary capillary permeability. To date, no literature has reported the effect of nootkatone on BALF parameters, and the present study provides the first evidence of its potential therapeutic benefit.

The effect of LPS on hematological parameters revealed a significant decrease in TEC, Hb, and PCV, along with a sharp increase in TLC in the DC group compared to the NC group. These significant changes in hematological parameters may be due to dysfunction of the hematopoietic system. The reduced TEC observed in the LPS-treated group might be attributed to enhanced lipid peroxidation, as evidenced by the significant increase in MDA concentration in the present study. Increased MDA levels may increase the susceptibility of RBCs to oxidative destruction, leading to anemia. The decrease in PCV% may also be due to an increased rate of RBC breakdown.

LPS instillation resulted in rapid and sustained neutrophilic airway inflammation, causing an elevated count of neutrophils and other leukocytes. The chemotactic action of various chemokines and cytokines secreted by activated macrophages at the site of injury further raises the risk of oxidative damage and promotes cellular lysis, ultimately contributing to the reduction in TEC, Hb, and PCV (34). In contrast, the NLD and NHD treatment groups showed significant improvements in hematological parameters throughout the experiment. The hemoprotective effects of nootkatone may be attributed to its free radical-scavenging ability and strong antioxidant properties.

The antioxidant profile in LPS-induced ALI was assessed. Glutathione (GSH) is a universal antioxidant that protects against exogenous toxic injury by strengthening defenses against ROS through free radical scavenging. It accomplishes this by directly donating a hydrogen atom and neutralizing free radicals. GSH is found in high concentrations in the epithelial lining of the lungs, where it protects against a wide range of inhaled oxidants. When GSH levels drop in tissues, cellular defenses against oxidative stress become impaired. The enzyme SOD catalyzes the dismutation of superoxide radicals into H_2_O_2_ or O_2,_ thereby preventing lipid peroxidation. It also plays a crucial protective role against tissue necrosis and is essential for defending against oxidative injury (35).

Catalase (CAT) is a vital enzyme in the cellular antioxidant defense system, as it catalyzes the decomposition of H_2_O_2_ into H_2_O and O_2_, thereby providing crucial protection against oxidative injury (36). In the present experiment, we found that LPS induces severe nitroso-oxidative stress in the pathogenesis of ALI, which might be due to the upregulation of inducible nitric oxide synthase (iNOS) and the consequent overproduction of nitric oxide (NO). Peroxynitrite, a key indicator of nitrosative stress, plays a vital role in the pathophysiology of acute conditions. Nitrite levels, which serve as an indirect measure of NO production, were found to be elevated due to increased nitric oxide synthase activity (37). MDA is generated as a degradation product during lipid peroxidation and serves as a reliable marker of oxidative damage.

Oxidative stress is a causative factor in a variety of clinical disorders, including airway inflammation, which contributes to the etiology and progression of ALI. In ALI or sepsis, there is a substantial increase in the release of different ROS, resulting in oxidative stress. The free radicals generated under these conditions play a significant role in the pathophysiology of sepsis-related oxidative injury (38). ROS attack the lipid layer of cell membranes, leading to the peroxidation of unsaturated fatty acids and the formation of highly cytotoxic compounds such as MDA, which compromise cellular stability. Furthermore, ROS deactivates key antioxidant enzymes such as SOD, GSH, and catalase (39, 40). The Nrf-2 is a key regulator of oxidative damage that, upon activation, upregulates antioxidant defense mechanisms, maintains cellular homeostasis, and modulates the MAPK pathway and the expression of NF-κB (41). The results of the present study indicate that the induction of ALI and subsequent lung tissue injury are responsible for the increased MDA levels and decreased GSH, SOD, and CAT levels observed (24, 42). Previous reports have shown that LPS administration leads to decreased CAT and SOD activity and increased MDA and nitrite levels, resulting in oxidative damage to lung tissue due to the overproduction of free radicals (43). In contrast, nootkatone treatment significantly elevated the expression of SOD, GSH, and CAT while reducing nitrite and MDA levels (44–46).

In the present study, cytokine levels were significantly higher in the DC group compared to the NC group. The activation of cytokines by LPS is well documented as a major contributor to severe lung injury and the initiation of strong inflammatory responses during the early stages of lung damage (47, 48). Inhibition of pro-inflammatory cytokines and their regulatory pathways have shown excellent success in reducing LPS-induced lung inflammation (47, 49). It has been demonstrated that cytokines play a crucial role in the pathophysiology of LPS-induced ALI (50, 51). LPS enhances signal transduction, leading to the activation of the NF-κB family of transcription factors in macrophages, which then promotes the production of a wide range of immunoregulatory and pro-inflammatory molecules (52). We evaluated the levels of key cytokines, including TNF-*α*, IL-6, IL-1β, IL-10, IL-17, IL-22, IFN-*γ*, and TGF-β1, which play central roles in the inflammatory cascade associated with LPS-induced acute lung injury (ALI). These mediators influence the integrity of the alveolar-capillary barrier, promote immune cell activation, and contribute to the accumulation of proteinaceous exudates. Among them, TGF-β1 is a critical fibrogenic cytokine involved in tissue remodeling and fibrosis. In our study, oropharyngeal administration of LPS markedly upregulated TGF-β1 expression, reflecting alveolar injury, while treatment with nootkatone significantly attenuated this response, indicating its potential anti-fibrotic properties. The innate immune organization is primarily accountable for the production of IL-6 during the acute phase of ALI, one of the first cytokines to be released. IL-6 has a number of immunoregulatory effects and can enhance the body’s overall immune response (53, 54). IL-1β is a pro-inflammatory cytokine primarily produced by macrophages and plays a key role in inflammation and immunomodulation (55). It is a major extracellular mediator of inflammation that often acts synergistically with TNF-α and is considered one of the central cytokines driving the inflammatory cytokine surge (21). TNF-α, another critical pro-inflammatory cytokine, can initiate the inflammatory cascade, damage vascular endothelial cells, and interact with its receptors in the lung tissues, causing enzyme leakage (56). According to reports, in LPS-induced murine ALI models, suppression of TNF-*α* and IL-1β effectively reduces pulmonary injury (57, 58). Innate immune cells, such as macrophages and other effector cells, also release IL-17 (59–61). IL-17 is known to promote neutrophil-mediated inflammation and may play a crucial role in modulating the inflammatory response during systemic inflammatory response syndrome (SIRS).

Neutrophils produce the anti-inflammatory cytokine IL-10, which plays a vital role as a negative regulator of SIRS by reducing the production of pro-inflammatory cytokines (61, 62). Conversely, IL-22 is primarily produced by activated Th1 and Th17 cells (63, 64), indicating that IL-22 functions as a distal modulator of the inflammatory response (65, 66). In the present study, nootkatone treatment resulted in decreased cytokine levels by inhibiting NF-κB activation and suppressing the transcription of pro-inflammatory cytokines. These results are consistent with previous reports supporting the anti-inflammatory effects of nootkatone (67, 68).

Compared to the NC group, the DC group showed markedly lower IL-10 levels. IL-10 is well known for its role in inhibiting macrophage and T-cell activation, as well as in suppressing the production of inflammatory cytokines. The decrease in IL-10 levels observed indicates the severity of lung injury (69). Previous *in vivo* studies have reported that IL-10–mediated protection of damaged lung tissue is most likely related to reduced neutrophil infiltration. In our study, the NHD group showed elevated IL-10 levels compared to the DC group. Earlier research has also reported that nootkatone exhibits anti-inflammatory properties against LPS-induced neuroinflammation (7).

The expression of inflammatory markers in LPS-induced ALI was investigated using immunohistochemistry. TNF-*α* acts as a ligand for TLR-4, initiating the NF-κB intracellular signaling pathway and triggering a cascade that leads to the release of cytokines and COX-2, which, in turn, enhances prostaglandin production and promotes inflammation (70). In ALI/ARDS, NF-κB serves as a key transcription factor involved in the progression of inflammation. Upon LPS entry into the cytoplasm, IκB becomes phosphorylated, allowing NF-κB to translocate into the nucleus, where it binds to specific promoter gene sequences and activates the transcription of pro-inflammatory mediators (51).

The anti-inflammatory impact of Nrf-2 has been linked to its crosstalk with the NF-κB transcription factor, a central regulator of inflammatory responses and several components of innate and adaptive immunity. Under typical physiological conditions, Nrf-2 remains inactive in the cytosol as part of the cell’s intrinsic antioxidant enzyme system. In our study, Nrf-2 expression was found to be significantly reduced in LPS-exposed animals compared to the NC group.

**Figure 11:**
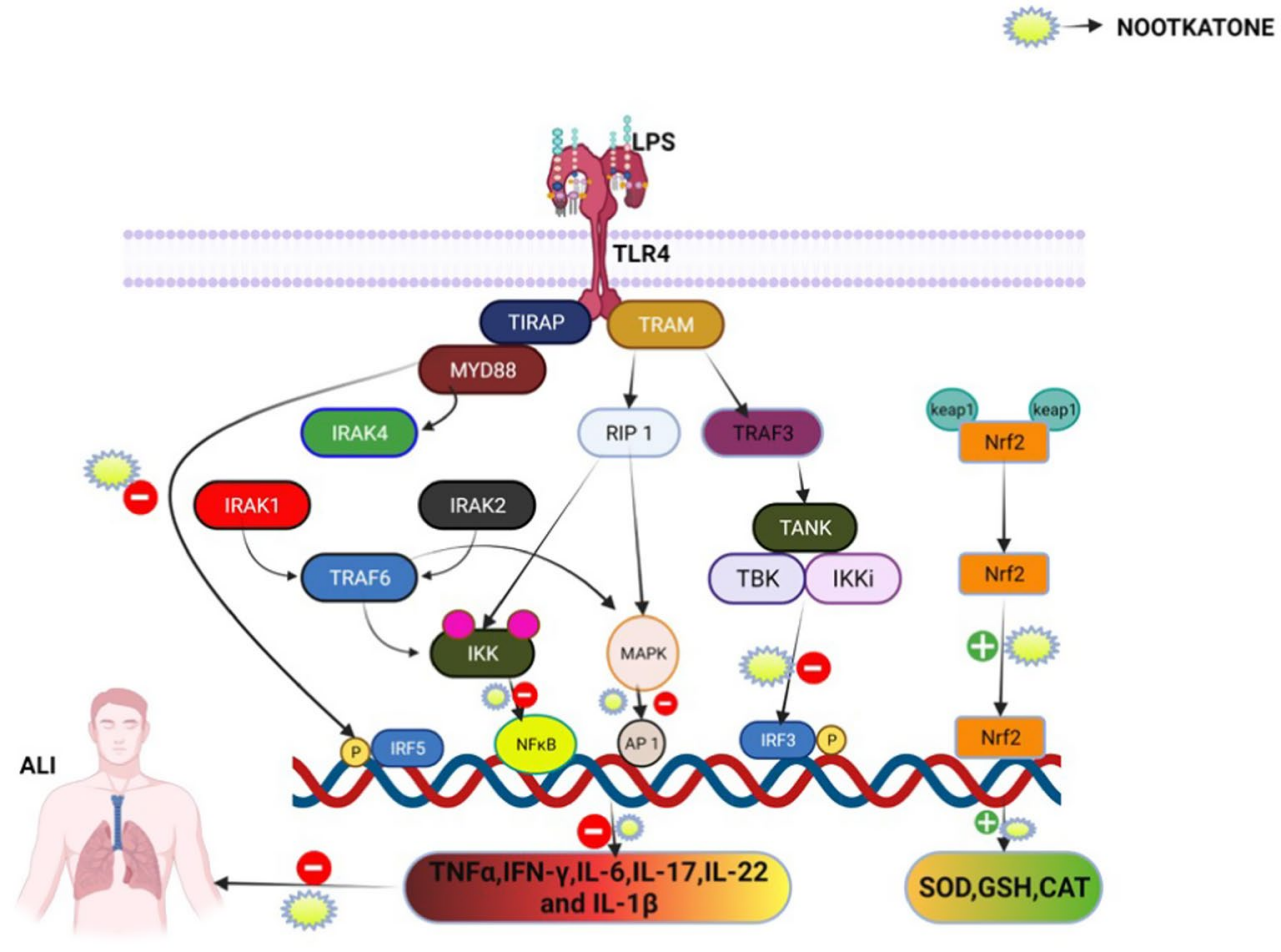
Nootkatone exerts a protective effect against LPS-induced acute lung injury (ALI). It modulates both inflammatory and oxidative stress pathways by inhibiting key components of the TLR4 signaling cascade, including IRAK1, TRAF6, MAPK, and NF-κB, thereby reducing the expression of pro-inflammatory cytokines. Simultaneously, nootkatone activates the Nrf2/Keap1 pathway, enhancing the expression of antioxidant enzymes such as SOD, GSH, and CAT. These dual actions contribute to the attenuation of lung inflammation and oxidative damage.

When the body experiences oxidative stress, Nrf-2 becomes activated and translocates into the nucleus, where it regulates genes involved in antioxidant defense and cellular protection (71). Figure 11 provides a hypothetical representation of the proposed mechanism of action of nootkatone. A recent study by Cui et al. (72) demonstrated that nootkatone protects against LPS-induced ALI by inhibiting the STING/TBK1/IRF3 pathway in alveolar macrophages, highlighting its role in modulating upstream innate immune signaling within a specific cell population. In contrast, our study is the first to demonstrate the modulation of the NF-κB/Nrf2 axis in ALI, characterized by the suppression of pro-inflammatory mediators (TNF-α, COX-2, IL-6, and IL-1β) and the activation of antioxidant responses (SOD, GSH, catalase, and HO-1).

In addition, our investigation extended beyond cytokine analysis to include oxidative stress markers, hematological indices, BALF cellularity, and histopathological scoring, thereby providing comprehensive organ-level validation. These findings suggest that the protective effects of nootkatone are mediated through its ability to modulate multiple inflammatory and oxidative signaling pathways. Beyond NF-κB inhibition and Nrf2 activation, previous studies have also shown that nootkatone activates AMPK, suppresses MAPK signaling, and improves mitochondrial function, thereby reducing ROS production and preserving energy homeostasis. Taken together, current evidence indicates that nootkatone exerts broad-spectrum anti-inflammatory and cytoprotective effects in ALI by targeting both upstream innate immune sensors and downstream transcriptional regulators.

In this study, nootkatone was administered as a pretreatment strategy to ensure systemic availability and to clearly establish its prophylactic efficacy. Although this approach may not fully represent post-injury treatment scenarios, it has translational relevance in veterinary medicine, where prophylactic interventions are commonly used in high-risk animals. Future studies should evaluate therapeutic administration after LPS exposure to more accurately reflect clinical practice.

### 4.1 Limitations

This study has certain limitations that need to be considered. Nootkatone was administered as a pretreatment, which may not fully reflect its therapeutic potential when administered after the onset of ALI. Therefore, future studies should examine its efficacy following post-LPS administration. Although this study demonstrated NF-κB downregulation and Nrf2 activation, more detailed molecular analyses are needed to confirm the downstream signaling pathways involved. In addition, the pharmacokinetics and lung tissue distribution of nootkatone were not evaluated. While immunohistochemistry provided supportive evidence, future investigations should incorporate higher-resolution imaging techniques and molecular assays to further substantiate these findings.

## 5 Conclusion

In summary, this study highlights the pharmacological potential of nootkatone in mitigating LPS-induced inflammation both *in vitro*, utilizing RAW 264.7 macrophages, and *in vivo*, specifically in ALI. LPS induces ALI by upregulating the NF-κB pathway (increasing the expression of pro-inflammatory cytokines), downregulating Nrf-2 (significantly altering oxidative stress parameters), and disrupting the alveolar epithelial–endothelial barrier, resulting in pulmonary edema and impaired lung function. Owing to its strong anti-inflammatory activity through the downregulation of the NF-κB pathway and the restoration of antioxidant capacity via upstream regulation of the Nrf-2 pathway, treatment with nootkatone effectively prevents LPS-induced ALI. These findings suggest that nootkatone holds promise in protecting against various forms of lung injury, including those associated with COVID-19. Future studies should further explore its therapeutic potential and underlying mechanisms to establish its clinical relevance.

## Data Availability

The original contributions presented in the study are included in the article/supplementary material, further inquiries can be directed to the corresponding author.
